# DNA repair genes in astrocytoma tumorigenesis, progression and therapy resistance

**DOI:** 10.1590/1678-4685-GMB-2019-0066

**Published:** 2019-12-13

**Authors:** Juliana Ferreira de Sousa, Rodolfo Bortolozo Serafim, Laura Marise de Freitas, Carla Raquel Fontana, Valeria Valente

**Affiliations:** 1 Radiation Oncology Branch, National Cancer Institute, National Institutes of Health, Bethesda, MD, U.S.A.; 2 Universidade de São Paulo, Faculdade de Medicina de Ribeirão Preto, Departamento de Biologia Celular e Molecular e Bioagentes Patogênicos, Universidade de São Paulo, Ribeirão Preto, SP, Brazil.; 3 Universidade de São Paulo, Instituto de Química, Departamento de Bioquímica, São Paulo, SP, Brazil.; 4 Universidade Estadual Paulista “Júlio de Mesquita Filho” (UNESP), Faculdade de Ciências Farmacêuticas, Departamento de Análises Clínicas, Araraquara, SP, Brazil.; 5 Centro de Terapia Celular (CEPID-FAPESP), Ribeirão Preto, SP, Brazil.

**Keywords:** Glioblastoma, DNA repair, biomarkers, tumor progression, therapy resistance

## Abstract

Glioblastoma (GBM) is the most common and malignant type of primary brain tumor, showing rapid development and resistance to therapies. On average, patients survive 14.6 months after diagnosis and less than 5% survive five years or more. Several pieces of evidence have suggested that the DNA damage signaling and repair activities are directly correlated with GBM phenotype and exhibit opposite functions in cancer establishment and progression. The functions of these pathways appear to present a dual role in tumorigenesis and cancer progression. Activation and/or overexpression of ATRX, ATM and RAD51 genes were extensively characterized as barriers for GBM initiation, but paradoxically the exacerbated activity of these genes was further associated with cancer progression to more aggressive stages. Excessive amounts of other DNA repair proteins, namely HJURP, EXO1, NEIL3, BRCA2, and BRIP, have also been connected to proliferative competence, resistance and poor prognosis. This scenario suggests that these networks help tumor cells to manage replicative stress and treatment-induced damage, diminishing genome instability and conferring therapy resistance. Finally, in this review we address promising new drugs and therapeutic approaches with potential to improve patient survival. However, despite all technological advances, the prognosis is still dismal and further research is needed to dissect such complex mechanisms.

## Introduction

Gliomas are brain cancers that present glial differentiation and represent a group of highly heterogeneous tumors with diverse histological, immunohistochemical and molecular characteristics (Louis *et al.*, 2016; Wirsching and Weller, 2016). They correspond to only 2% of the overall cases of cancer. However, despite the low incidence, these cancers represent an important cause of death due to the elevated mortality associated, especially regarding the most malignant and common form, glioblastoma (GBM). Patients diagnosed with a GBM present an average survival of 14.6 months and only 5% survives for more than 5 years (McNeill, 2016). GBM is characterized by prominent dedifferentiation, diffuse infiltration, exacerbated proliferation, presence of necrosis and angiogenesis, resistance to apoptosis and conspicuous genomic instability. These tumors are seriously aggressive and resistant to available treatments, which involve surgical resection, radiotherapy, and chemotherapy with temozolomide (TMZ) (Louis *et al.*, 2016). In this review, we focused on alterations of DNA damage response (DDR) and DNA repair genes encountered in astrocytomas from different grades, aiming to draw an integrated view of how dysfunctions in these pieces of machinery are orchestrated to allow tumorigenesis, cancer progression, resistance to therapy, as well as its potential involvement in controlling the marked genomic instability of GBM cells. Also, we suggest some future perspectives of promising approaches that could possibly improve GBM treatment.

## Glioma classification and frequent genetic alterations

The first complete and robust classification of the tumors from the central nervous system (CNS) proposed by the World Health Organization (WHO) was published in 2007 (Louis *et al.*, 2007). This publication redesigned the previous grading system (Kernohan and Mabon, 1949), as follows: grade I was defined as benign tumors with low proliferative potential that can be cured after surgical resection; grade II referred to lesions with moderate mitotic activity, infiltrative capability and tendency to progress to higher grades of malignancy; grade III correspond to tumors with histological evidence of malignancy, as nuclear atypia and high mitotic activity; and grade IV that present higher levels of atypia, exacerbated mitotic activity, besides angiogenesis and necrosis, which are associated with rapid tumor growth and fatal outcome for patients. In summary, the assortment was largely based on differentiation levels and histopathological features (Louis *et al.*, 2007).

More recently, in 2016, the newest edition of the WHO classification was published (Louis *et al.*, 2016), integrating genetic features and novel molecular biomarkers with the traditional histology examination. The current update changed grouping criteria, redefined diffuse gliomas, included new entities and discouraged the diagnosis of tumors difficult to be defined, such as oligoastrocytomas. Three major types of gliomas were distinguished, diffuse astrocytic and oligodendroglial, other astrocytic, and ependymal tumors. Grade I to IV assignment as a malignancy ruler was kept and mutations in IDH1 and histone H3 (H3K27M) were included, as well as the 1p/19q-codeletion (Table 1) (Louis *et al.*, 2007, 2016). Moreover, grade II tumors were considered low-grade glioma (LGG) due to their less aggressive behavior, and grades III and IV as high grade (HGG), as they present worse prognoses (Louis *et al.*, 2016). Considering that approximately 76% of all gliomas exhibit astrocytic origin (Louis *et al.*, 2016; McNeill, 2016), hereafter we use the malignancy scale and LGG and HGG to refer only to astrocytoma.

**Table 1:** WHO 2016 types and grade of glioma.

Diffuse astrocytic and oligodentroglial tumors	Grade
Diffuse astrocytoma, IDH-mutant	II
anaplastic astrocytoma, IDH-mutant	III
glioblastoma, IDH-wildtype	IV
glioblastoma, IDH-mutant	IV
diffuse midline glioma, H3K27M-mutant	IV
oligodendroglioma, IDH mutant and 1p/19q-codeleted	II
Anaplastic oligodendroglioma, IDH-mutant and 1p/19q-codeleted	III
**Other astrocitic tumors**	
Pilocytic astrocytoma	I
Subependymal giant cell astrocytoma	I
Pleomorphic xanthoastrocytoma	II
anaplastic pleomorphic xanthoastrocytoma	III
**Ependymal tumors**	
Subependymoma	I
myxopapillary ependymoma	I
ependymoma	II
ependymoma, RELA fusion-positive	II or III
Anaplastic ependymoma	III

LGG usually present an indolent behavior, but about 70% of cases undergo progression to grades III and IV within 5 to 10 years after diagnosis. Occurring mainly in childhood, LGG represents more than 30% of central nervous system neoplasms in this population (Louis *et al.*, 2016). Despite the typical heterogeneity, LGG harbor alterations in the *BRAF* gene that commonly lead to the loss of its regulatory N-terminal region. Other genetic abnormalities are also described, but in all cases, the defects frequently lead to constitutive activation of the MAP (mitogen-activated protein) kinase pathway (Jones *et al.*, 2012; Zhang *et al.*, 2013). Besides *BRAF* mutations, translocations involving tyrosine kinase receptors have been likewise documented. For example, neurotrophic tyrosine kinase receptors (*NTRK*) 2 and 3 were found fused by its N-terminus with other genes, acquiring the ability to interact with actin or topoisomerase I. Interestingly, *NTRK* fusions have also been noticed in pediatric HGG (Wu *et al.*, 2014), suggesting a potential general role for these types of fusions in glioma development. Furthermore, mutations in fibroblast growth factor receptor 1 (*FGFR1*) are the second most common point mutation in LGG, after *BRAF* V600E (Jones *et al.*, 2013).

Glioblastoma (astrocytoma grade IV) is the most common and aggressive HGG, accounting for 16% of brain tumors and 60-75% of astrocytomas (Thakkar *et al.*, 2014). GBM occurs mainly in elderly individuals among 45-75 years of age, usually leading the patient to death in 12-15 months after diagnosis. Even under rigorous therapy, the majority of cases relapses in 1-2 years after surgery and less than 5% of patients survive for 5 years or more (McNeill, 2016). GBM is further classified as primary or secondary according to their clinical history. Primary GBM occurs in a *de novo* manner without evidence of previous lesion and accounts for 90% of cases; secondary GBM is a result of LGG progression into HGG and represents 10% of cases (Ohgaki and Kleihues, 2013; Louis *et al.*, 2014). Primary and secondary GBMs present marked genetic differences and distinct transcriptional activity that identify unique entities, predict prognosis and delineate a progression pattern (Maher *et al.*, 2006; Ohgaki and Kleihues, 2013).

The Cancer Genome Atlas (TCGA) Research Network performed detailed genome-wide analyses and disclosed the intricate genetic profile of GBMs, and grade II and III gliomas, by characterizing more than 1000 human samples. The majority of cases harbor alterations in the following genes: *MGMT, IDH1, TP53, RB1, RTK, RAS, EGFR, cyclin D1/3, MDM2, PTEN, CDK4, PDGFRA, PIK3CA, NF1, PIK3R1, LZTR1, BRAF, FGFR1, FGFR2, FGFR3, ATRX, TERT, NOTCH1, FUBP1, CIC* (Cancer Genome Atlas Research Network, 2008, 2015). Considering the landscape of alterations characterized, three core signaling pathways underlying GBM pathogenesis were identified: tyrosine kinase receptors, p53, and retinoblastoma. Additionally, global transcriptional profiling allowed a more refined classification of GBMs into four molecularly distinct subgroups: proneural, neural, classical and mesenchymal that are also characterized by a particular set of high frequent mutations (Table 2) (Verhaak *et al.*, 2010; Brennan *et al.*, 2013). It was also characterized a subtype of proneural GBMs that presents a hypermethylated phenotype of CpG islands (G-CIMP), which is associated with improved survival and is more prevalent in LGG (Noushmehr *et al.*, 2010; Brennan *et al.*, 2013). The response to the different therapy protocols currently applied varies considerably among these transcriptional subgroups. Classical and mesenchymal subtypes obtain benefit from more intensive treatment, while patients with the neural profile apparently get only a small increase in survival and the proneural show no increment (Verhaak *et al.*, 2010). However, even for patients who benefit from intensive therapy, the survival gain corresponds to a few months only, and to the best of our knowledge, there is no literature evidence of the clinical use of the subclassification.

**Table 2:** GBM subgroups and their main genetic changes.

Classical	Mesenchymal	Neural	Proneural
EGFR mutation/overexpression	NF1 loss/mutation	EGFR overexpression	PDGFRA amplification
PTEN loss/mutation	TP53 loss/mutation	neuron markers expression	IDH1 mutation
CDKN2A loss	PTEN loss/mutation		PIK3A/PIK3R1 mutations
NES overexpression	MET, CHI3L1, CD44, MERTK overexpression		TP53, CDKN2A, PTEN loss/mutation
Notch and Shh pathways activation	TNF family and NFkB pathways activation		proneural markers expression

Despite the diversity of genetic alterations underlying GBM pathogenesis, all subtypes present remarkable proliferation rate, diffuse infiltration, enhanced survival capacity and robust angiogenesis, which provide high resistance to the available therapies and unavoidable recurrence. All of these characteristics added to prominent intra-tumor heterogeneity and genomic instability, make GBM one of the most complex types of cancer frequently associated with dismal prognosis (Noushmehr *et al.*, 2010; Verhaak *et al.*, 2010; Brennan *et al.*, 2013). The robust characterization of gliomas now available, encompassing large-scale genetic and epigenetic profiling, high throughput transcriptomic and proteomic analysis, revealed novel important GBM features, as new biomarkers and unique signatures capable of providing better diagnosis, predict prognosis and/or treatment response (Cancer Genome Atlas Research Network, 2008; Noushmehr *et al.*, 2010). The list of significant biomarkers is growing in an astonishing manner and includes a wide range of molecules such as lncRNAs and microRNAs (e.g. HOTAIR and miR-141), and several DNA repair genes (Boccard *et al.*, 2015; Bian *et al.*, 2016; Reon *et al.*, 2016), which are importantly correlated with genome stability and will be explored in more detail in the next sections. Table 3 summarizes the main DNA repair biomarkers discussed in this review.

**Table 3:** DNA repair genes considered biomarkers* of GBM susceptility and/or progression.

Gene	Alteration	Impact on disease progression	References
MGMT	promoter methylation	response to TMZ treatment	Hegi *et al.*, 2005; Hegi *et al.*, 2008; Stupp *et al.*, 2009; Binabaj *et al.*, 2018
APNG	overexpression	controversial	Agnihotri *et al.*, 2012; Fosmark *et al.*, 2017
HJURP	overexpression	poor outcome, worse overall survival	de Tayrac *et al.*, 2011; Valente *et al.*, 2013
DDB2	reduction	reduced survival	de Sousa *et al.*, 2017
BRCA2	overexpression	reduced survival	de Sousa *et al.*, 2017
BRIP1	overexpression	reduced survival	de Sousa *et al.*, 2017
XRCC3	polymorphism	increased GBM susceptibility	Custodio *et al.*, 2012
EXO1	polymorphism	increased GBM susceptibility	Chang *et al.*, 2008
EXO1	overexpression	reduced survival	de Sousa *et al.*, 2017
NEIL3	overexpression	reduced survival	de Sousa *et al.*, 2017
MSH6	mutations	controversial	Hunter *et al.*, 2006; Maxwell *et al.*, 2008; Yip *et al.*, 2009; Sun *et al.*, 2018

^*^This table shows only the biomarkers discussed throughout the review. Please refer to the text for further information.

## DNA repair genes as biomarkers of astrocytoma aggressiveness

The methylation status of *MGMT* (O-6-Methylguanine-DNA-Methyltransferase) promoter was the first biomarker to be used for patient stratification in clinical trials as a predictor of GBM response to treatment with alkylating agents (Hegi *et al.*, 2005, 2008). The *MGMT* gene encodes a DNA repair protein responsible for the removal of alkylation at guanines O6 position, a site that is commonly altered by TMZ, the gold standard chemotherapeutic for GBM treatment. Methylation of the MGMT promoter reduces protein expression, thus impairing the repair capacity of TMZ-induced damage, boosting the response to treatment (Hegi *et al.*, 2008).

In a randomized phase III clinical trial with a set of 206 GBM patients, Stupp and colleagues observed that 45% of patients presented methylations in the *MGMT* promoter. This feature was associated with a better overall survival, 21.7 months after chemotherapy associated with radiotherapy, in comparison to 15.3 months for patients carrying non-methylated genotype (Stupp *et al.*, 2009). More recently, a meta-analysis of 10 eligible studies, including the MGMT methylation status of more than four thousand subjects, confirmed that patients bearing this genotype present longer overall survival (Binabaj *et al.*, 2018), emphasizing MGMT status as an independent indicator of a favorable prognosis. *MGMT* methylation could also be found in patient serum and strongly correlated with its presence in the tumor tissues (Fiano *et al.*, 2014), suggesting that detection in blood samples could represent a reliable tool to predict response to TMZ treatment. *MGMT* methylation also disclosed its relevance as biomarker for other types of malignancies, including breast (Neto *et al.*, 2012), colorectal (Lee *et al.*, 2009), prostate (Cortese *et al.*, 2012), cervical (Sun *et al.*, 2015), gastric (Jin *et al.*, 2014) and lung (Ostrow *et al.*, 2010) cancers.

Therefore, MGMT promoter methylation, i.e., reduced MGMT protein expression, is a frequent epigenetic alteration in GBM patients related to better outcome, survival and response to treatment. On the other hand, when MGMT promoter is not methylated, i.e. normal MGMT expression, this protein is considered a good therapeutic target, once it is available and susceptible to pharmacological inhibition. Hence, several groups are concentrating efforts to develop strategies to reduce MGMT activity and/or expression, enhancing TMZ sensitivity.

Additionally, among patients carrying the *MGMT* methylated phenotype, those with high levels of the alkyl purine-DNA-N-glycosylase (APNG) enzyme present better overall survival and this result was supported by data from TCGA database (Fosmark *et al.*, 2017), making the APNG expression levels an important factor to be associated to *MGMT* methylation status. APNG is a DNA repair enzyme involved in the base excision repair (BER) pathway, which is responsible for removing methyl of adducts, induced by alkylating agents, creating apurinic or apyrimidinic sites (Evans *et al.*, 2000). Curiously, APNG overexpression was also associated with poor survival (Agnihotri *et al.*, 2012), suggesting a controversial role for this enzyme as a prognostic biomarker. Taken together, these pieces of evidence highlight the importance of complementary studies toward the development of different therapy approaches and novel drugs that do not rely exclusively on *MGMT* methylation phenotype.

Expression levels of the Holiday Junction Recognizing Protein (HJURP) were also correlated with prognosis of astrocytoma patients. HJURP was reported as highly overexpressed in tumors from different grades and showed an independent capacity of survival prediction (Valente *et al.*, 2009, 2013). HJURP was also shown to be involved in DNA double-strand breaks (DSB) restoration (Kato *et al.*, 2007) by mechanisms not yet characterized. It has also important roles in the deposition of Centromere Protein A (CENP-A) at the centromeric chromatin (Foltz *et al.*, 2009), presenting capacity to allow centromeric chromatin expansion (Perpelescu *et al.*, 2015) and assembly of ectopic kinetochores (Barnhart *et al.*, 2011). Other studies have described the association between *HJURP* overexpression, combined with additional alterations, and a higher risk of death for GBM patients (de Tayrac *et al.*, 2011; Valente *et al.*, 2013). Moreover, it was also reported that *HJURP* overexpression has an independent prognostic value for breast (Hu *et al.*, 2010), lung (Kato *et al.*, 2007), liver (Hu *et al.*, 2017) and ovarian (Li *et al.*, 2018) cancer patients, reinforcing the involvement of this protein with cancer aggressiveness and poor outcome.

Furthermore, several expression signatures of DNA repair genes were strongly associated with poor prognosis of astrocytoma patients. Among the alterations included in these signatures, reduction of *DDB2* and overexpression of *EXO1*, *NEIL3*, *BRCA2* and *BRIP1,* were independently correlated with worse prognoses, revealing single-gene signatures that represent new feasible biomarkers. *EXO1* and *NEIL3* exhibited remarkable overexpression and showed to be involved in DSB restoration kinetics and radiation resistance of GBM cell lines, respectively (de Sousa *et al.*, 2017). Additional studies are necessary to better describe their roles in GBM biology and potential enrollment as biomarkers. In agreement, a study including 539 GBM cases identified and validated a gene expression signature including 15 key DNA repair genes significantly correlated to prognosis, and five of them (*CDK7*, *DDB2*, *RNH1*, *RFC2* and *FAH*) were highly predictive of recurrence and disease-free survival (Kun *et al.*, 2017).

In a recent study, an expression profiling of selected 154 genes involved in DNA damage signaling/repair and cell cycle was accomplished in cohorts containing paired samples of primary and recurrent GBM. Gobin *et al.* (2019) identified and validated a 27-gene signature that was able to stratify patients in two well-defined groups (G1 and G3) showing co-regulation and inverse expression patterns. A third subset containing samples with a more neutral profile formed a separate group named G2. Although no correlation with prognosis was found when only primary or paired GBM cohorts were considered, when analyzing only the cases of recurrence, the progression-free and overall survival were significantly worse in patients whose tumors progressed from G3 to G1 profile. Additionally, the use of inhibitors targeting RAD51 and mitotic kinases in tumor-derived cell cultures promoted a decrease in the viability of G3 cells. These data suggested that specific targets, selected on the basis of prognosis-correlated signatures, might represent vulnerabilities of a subset of tumors and can provide guidelines for personalized therapies (Gobin *et al.*, 2019).

## DNA repair functions in tumorigenesis and progression

**Figura 1:**
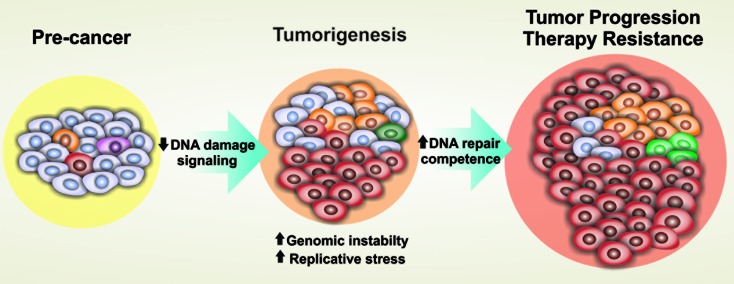
DNA damage signaling and repair pathways show opposite regulation in tumorigenesis and tumor progression. Early in tumorigenesis, oncogene activation leads to replication stress and DNA damage, usually triggering the DDR machinery and leading to checkpoint-imposed senescence or cell death. When this barrier is overcome, by loss-of-function mutations in DDR and/or DNA repair genes, tumor establishment ensues. Progressively advanced tumors experience increasing levels of replication stress and genetic instability and often adapt to this environment, developing exacerbated DNA repair competences that avoid cell death and favor tumor progression.

The DNA damage response (DDR) and the downstream recruited DNA repair machinery cross-communicate to form an intricate network of genome surveillance that identifies and repairs DNA injuries, protecting cells from intrinsic and microenvironmental genotoxic stress. Increasing pieces of evidence have been showing that this system presents antagonistic roles in tumorigenesis and tumor progression. Several studies have demonstrated that DDR and DNA repair genes may be inactivated in early tumorigenesis, enabling genomic instability and tumor development (Golmard *et al.*, 2013; Nissar *et al.*, 2014), whereas secondary mutations grant a selective advantage to the tumor (Gorgoulis *et al.*, 2005). Once the tumor is established, the repair activity undergoes reactivation, which avoids cell collapsing and allows tumor progression (Kauffmann *et al.*, 2008; Turner *et al.*, 2015), and is also associated with resistance to treatments (Eich *et al.*, 2013; Atkins *et al.*, 2015) (Figure 1).

Several studies have demonstrated that activation of DDR-DNA repair system works as an oncogene-induced barrier against tumor establishment (Bartek *et al.*, 2007; Squatrito and Holland, 2011), and mutations or alterations that lead to loss of function or downregulation could represent a trigger for gliomagenesis (Cha and Yim, 2013). In a study using a mouse model for glioma development, it was demonstrated that the induced expression of RAD51, a central protein for repair by homologous recombination (HR), decreases both the incidence of oncogene-induced glioma and the genomic instability, impairing carcinogenesis (Westermark *et al.*, 2011). Using a similar model, Squatrito and colleagues showed that components of the DDR pathway are frequently altered in gliomas and loss of ATM or its downstream targets accelerates tumor formation (Squatrito *et al.*, 2010). Loss of ATRX, a protein required for non-homologous end joining (NHEJ), was also reported to promote GBM growth in an animal model (Koschmann *et al.*, 2016).

A study conducted within the Brazilian population showed that patients carrying the Thr241Met polymorphism in the *XRCC3* gene presented an increased risk of tumor development, suggesting that its malfunction contributes to astrocytoma and glioblastoma susceptibility. *XRCC3* encodes a protein involved in HR repair, and this polymorphism can potentially affect the enzyme function as well as its interaction with other repair proteins (Custodio *et al.*, 2012). Additionally, a nonsynonymous single nucleotide polymorphism (SNP) in the *EXO1* gene was found to be potentially associated with GBM susceptibility. *EXO1* is an important exonuclease of HR and the evaluated SNP promotes a drastic amino acid change that could affect the protein internal structure, as well as its protein-protein binding interface, impairing its normal function (Chang *et al.*, 2008). Other polymorphisms of this nature have been also associated with GBM risk (Franceschi *et al.*, 2016; Qi *et al.*, 2016).

Paradoxically, DDR and the repair machinery act as a double-edged sword during tumorigenesis and cancer progression. Once the tumor has been established, replicative stress can promote the aberrant constitutive activation of DDR and repair execution (Bartkova *et al.*, 2010; Carruthers *et al.*, 2018), allowing tumor progression and driving treatment resistance. In turn, the exacerbated activity of these pathways defend malignantly transformed cells from replicative stress, high mutation rates, and the rampant genome instability (Kauffmann *et al.*, 2008; Turner *et al.*, 2015). Bartkova and colleagues observed that DDR is constitutively active in LGG and GBM samples, but not in normal brain tissues nor in regions adjacent to the tumor. Interestingly, in GBMs, which present the highest proliferation rates, the amounts of DNA damage detected were diminished in comparison to LGG (Bartkova *et al.*, 2010). This data suggest that the DDR machinery is more effective in GBM than in LGG, helping highly malignant cells to manage their unstable genome and avoid collapse and death. Moreover, an increase in NHEJ and HR activities, mediated by the RTK/RAS pathway, was observed along with glioma progression (Turner *et al.*, 2015).

More recently, *EXO1* and *NEIL3*, DNA repair genes extremely overexpressed in different grade astrocytomas, showed a strong correlation with patient survival and GBM cells viability (de Sousa *et al.*, 2017). EXO1 is a 5’ to 3’ exonuclease that resects the blunt ends of DSBs generating the single-strand tail necessary to invade the double-strand DNA used as a template in HR repair (Kim and Wilson, 2012). Silencing of *EXO1* in T98G cells led to faster restoration of DNA injury induced by ionizing radiation (IR), suggesting that the absence of *EXO1* possibly directs the DSB repair to the faster and error-prone NHEJ pathway (de Sousa *et al.*, 2017). These results indicate a potential role of EXO1 to facilitate DNA repair during astrocytoma progression. Those data are in agreement with the increase in NHEJ and HR activities during glioma progression observed by Turner *et al.* (2015).

Additionally, *NEIL3* knockdown was associated with a higher percentage of DNA damage and cell death after IR (de Sousa *et al.*, 2017). *NEIL3* is a DNA glycosidase that participates of BER by removing oxidized bases, which can be induced secondarily by IR, giving rise to apurinic/apyrimidinic sites that are recognized and converted to single-strand breaks (SSB) by the endonuclease APEX2 (Takao *et al.*, 2009). These observations suggested a potential role for *NEIL3* in the management of oxidative stress, supporting tumor progression. Taken together, these data imply the progressive requirement of DDR signaling and enhanced DNA repair competence accompanying tumor progression.

Along with progression, malignant cells usually acquire genetic alterations that enable metastasis and several models have been proposed to clarify how this intricate process occurs (Hunter, 2015). It is well known that GBM cells are highly infiltrative and relapse mainly locally, but they can also easily migrate and spread along nerves, meninges, and local blood vessels, inducing CNS metastasis (Scherer, 1938; Awan *et al.*, 2015). The interactions between endothelial and GBM cells in microenvironmental niches seem to be important for progression and dispersion (Gilbertson and Rich, 2007). However, less than 2% of GBM cases metastasize outside the CNS (Beauchesne, 2011) and the roles of DNA repair genes during metastasis onset are controversial. In melanoma, for example, the upregulation of DNA repair genes is related to metastasis, while in many other tumors the opposite has been reported (Broustas and Lieberman, 2014).

## DNA repair and GBM resistance to treatment

**Figura 2:**
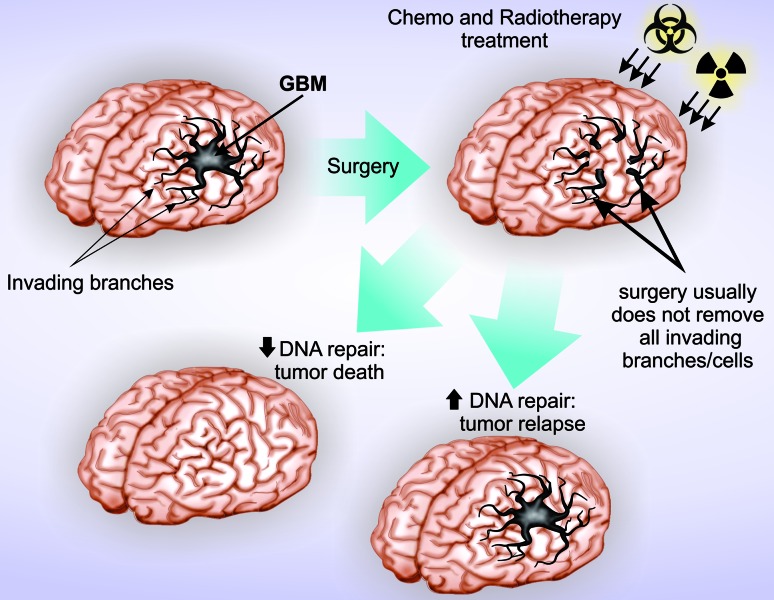
DNA repair competences predispose GBM to treatment resistance. The current standard treatment for GBM consists of surgery followed by chemoradiotherapy. Surgery frequently presents low efficiency due to the invasive nature of GBM cells, making difficult the complete resection. Tumors cells with higher DNA repair capabilities can efficiently manage chemoradiotherapy-induced DNA damage and support subsequent tumor relapse. Successful treatment is attainable when surgery can safely remove at least 75% of tumor tissue and the remaining cells present low DNA repair activity.

The therapy employed for GBM patients is usually multimodal and involves surgical resection, as much as considered safe, followed by adjuvant chemo and radiotherapy (CRT). However, the specificities of the therapeutic protocols are established according to each case’s necessity. The best available protocol for GBM treatment is the one reported by Stupp and colleagues, which indicates surgery followed by 6 weeks of CRT in tumor bed plus 6 additional cycles of TMZ-only (Stupp *et al.*, 2005). This treatment reduces death risk by 37% but survival prolongation is still minimal due to the high resistance of GBM cells and frequent recurrence (Stupp *et al.*, 2005, 2009) (Figure 2), emphasizing the urgency of better disease control and improvement of patient’s survival and life quality. In this section, we exploit literature that suggest the enrollment of the exacerbated activity of DNA repair pathways in treatment resistance, as well as their potential roles in subpopulations of cells that drive tumor relapse.

MGMT is the most studied DNA repair protein regarding associations with treatment response. The methylation status of the *MGMT* promoter affects protein expression and directly modulates TMZ response (Hegi *et al.*, 2005, 2008). However, some patients did not present any clinical improvement after TMZ administration, even when MGMT levels are reduced, indicating that methylated *MGMT* promoter uniquely does not mean a successful treatment (Hegi *et al.*, 2005). Additionally, it is also known that TMZ triggers the activity of different repair pathways, such as, BER (Yoshimoto *et al.*, 2012), MMR, NER, NHEJ and HR (Nagel *et al.*, 2017), which may also be considered to predict resistance.

Moreover, N-methylated bases are not recognized by MGMT and trigger BER pathway activation, suggesting that BER machinery could be targeted to enhance TMZ effectiveness. In fact, the inhibition of APNG, promoted a decrease in TMZ resistance, even in MGMT competent cells. It was also shown that the disruption of Ape1 (Bobola *et al.*, 2001) or DNA polymerase β (Trivedi *et al.*, 2008) activity sensitizes cells to TMZ. Moreover, increased NER activity was detected after treatment with TMZ (Nagel *et al.*, 2017), while downregulation of *ERCC1* promoted higher sensitivity to TMZ treatment, similarly to MGMT inhibition (Boccard *et al.*, 2015).

MMR machinery defects have also been associated with TMZ resistance. The MMR pathway acts primarily during replication, recognizing and correcting insertions, deletions and base misincorporations, such as the mismatch between a TMZ-induced O6-methylguanine (O6-MeG) and thymidine. However, only daughter strands can undergo this type of repair and, consequently, the misincorporated thymidine is removed but the O6-MeG remains on the template strand. This mechanism occurs repeatedly and produces a futile cycle of repair, generates DSBs, activates ATR and Chk1, and ultimately can cause cell cycle arrest and cell death (Zhang *et al.*, 2012). Thus, cells defective in MMR do not recognize DNA lesions and evade cell cycle arrest. Reduction of MSH6, a protein involved in mismatch recognition and MMR initiation, increased TMZ resistance of A172TR3 cells, which primarily exhibited a resistant phenotype (Yip *et al.*, 2009). Additionally, clinical data showed the presence of *MSH6* mutations in GBM specimens after TMZ therapy but not in pre-treatment samples, suggesting that these changes results from CT and may contribute to recurrence (Hunter *et al.*, 2006; Yip *et al.*, 2009). However, other studies suggest that MMR deficiency does not mediate clinical resistance to temozolomide (Maxwell *et al.*, 2008) and that the upregulation of MSH6 could be associated with resistance, opposing the relationship between MMR and TMZ resistance (Sun *et al.*, 2018). Thus, further studies are necessary to better elucidate the complex network involved in DNA repair and its relationship TMZ resistance.

Two major pathways, NHEJ and HR, are responsible for the DSBs repair. In contrast to NHEJ, which works at any moment of the cell cycle, HR pathway acts preferentially during S and G2 phases, when sister chromatids are available to be used as a repair template. HR restores DSBs generated by IR, as well as those produced by replication fork collapse (Helleday, 2010). Although TMZ does not cause DSB directly, O6-MeG can lead to futile repair cycles, stalling replication forks and inducing DSBs subsequently (Roos and Kaina, 2013). Once TMZ can produce DSB during S phase, HR is more relevant than NHEJ to repair these lesions. It has been demonstrated that mutation or knockdown of ATM or ATR kinases, which are sensors of DSB occurrence, sensitizes cells to TMZ (Eich *et al.*, 2013). Moreover, the pharmacological inhibition or knockdown of downstream proteins in the HR pathway, such as BRCA2, RAD51, and CHK2, also increased sensitivity to treatment (Quiros *et al.*, 2011; Eich *et al.*, 2013). Furthermore, GBM cells treated with other alkylating agents were more sensitive after inhibition of MRE11, a component of MRN complex that recognizes DNA lesions and recruits ATM kinase (Berte *et al.*, 2016). In contrast, some reports suggest that ATM/ATR activation is required to induce cell death after the MMR futile cycle is established (Caporali *et al.*, 2004) and that MRE11 knockdown decreases sensitivity to TMZ (Mirzoeva *et al.*, 2006). Altogether, these data highlight the complexity of responses to treatment and imply that the whole genetic background must be considered to better predict therapy efficiency.

IR causes several types of DNA damage and is frequently employed as a complementary therapy to GBM patients. Several studies have suggested that RAD51 (HR) and DNA-PKcs (NHEJ) activities may be related to GBM resistance to IR (Short *et al.*, 2011; King *et al.*, 2017). Russell and colleagues showed that GBM cells pretreated with Gleevec, an indirect inhibitor of RAD51, presented a reduction in RAD51 foci formation and higher sensitivity to IR (Russell *et al.*, 2003). Likewise, GBM cells with DNA-PKcs deficiency or inhibition showed suppression of IR-induced migration, invasion, and microvascular formation and presented higher levels cell death (Zhuang *et al.*, 2011; Gustafsson *et al.*, 2014; Liu *et al.*, 2015). Taken together, these data suggest that NHEJ and HR inhibitory agents present a great therapeutic potential.

Poly ADP-ribose polymerase 1 (PARP1) is a nuclear enzyme crucial for the overall repair process. PARP1 is rapidly activated by strand breaks and signals the presence of DNA lesions by attaching ADP-ribose units to chromatin proteins, leading to the recruitment of the downstream targets (Kim *et al.*, 2005; Luo and Kraus, 2012). In DSB repair, PARP1 has been shown to be responsible for the recruitment of MRE11 and NBS1 (Haince *et al.*, 2008), which are essential for both HR and NHEJ pathways (Roos and Krumm, 2016). Therefore, pharmacological inhibition of PARP1 may represent an excellent adjuvant therapy, since its inhibition could sensitize GBM cells to TMZ and IR, even when MGMT is normally expressed or MMR is deficient (Tentori *et al.*, 2002; Barazzuol *et al.*, 2013). Indeed, using *MGMT* non-methylated GBM models, a recent study showed that the association of veliparib, a PARP1 inhibitor, with IR inhibited colony formation, reduced the levels of MRE11 (HR pathway) and increased apoptosis. Furthermore, the oral administration of veliparib plus concomitant RT induced apoptosis and diminished cell proliferation in mice (Jue *et al.*, 2017).

Additionally, inhibition of PARP1 intensifies SSBs occurrence, which are converted to DSBs during replication. This effect is greater in *BRCA1/2* defective cells because missed DSBs restoration is secondly impaired and cell death is activated (Helleday, 2011). A great variety of studies and clinical trials have been conducted to evaluate the safety and efficacy of several PARP1 inhibitors (PARPi) exploiting its synthetic lethality in *BRCA*-defective tumors, mostly in breast and ovarian cancer (Yap *et al.*, 2019). Although mutations in *BRCA* genes are rare in GBM, *PTEN* mutations similarly impair HR and are found in one-third of cases, enabling the exploration of PARPi synthetic lethality also for GBM (McEllin *et al.*, 2010). However, the literature is still not conclusive about the benefits of the use of PARPi for GBM patients (Gupta *et al.*, 2018). There are *in vivo*/*in vitro* conflicting data (Gupta *et al.*, 2014) and PARPi have limited permeability and an efflux liability through the blood-brain barrier, showing heterogeneous response (Sarkaria *et al.*, 2018). So far, the inhibition of constitutively active DDR and repair proteins, a common alteration in GBM, shows great potential to improve treatment effectiveness.

The high resistance of GBM to therapies has also been associated with the presence of cancer stem cells, which differ from other GBM cells by being capable of unlimited self-renewal and presenting low proliferative ratios. These characteristics certainly contribute to resistance once usual treatments aim to eliminate highly proliferative cells, making this population very relevant to clinical treatment. GBM cancer stem cells (CSC) subpopulations can be identified by the expression of *CD133*, *SOX-2* and *Nestin* (Colleoni and Torrente, 2008; Ma *et al.*, 2008) that were all negatively correlated with patient survival (Zeppernick *et al.*, 2008), highlighting the importance to uncover mechanisms underlying CSCs competences.

Recently, Carruthers and coworkers showed that GBM CSCs present constitutively high levels of replicative stress, both *in vitro* and *in vivo*, and provided evidence that this phenotype underlies the activation of DDR and consequent radiation resistance (Carruthers *et al.*, 2018). In agreement, recent studies have demonstrated that GBM CSCs exhibit greater efficiency in the activation of DNA damage sensors, as ATM, 53BP1 and H2AX, and are more resistant to CT. These cells can also become dormant in drug presence and usually restart proliferation after drug withdrawal (Annovazzi *et al.*, 2015). In addition, inhibition of RAD51 in glioma stem cells reduced IR-induced foci formation and DSB repair, diminishing CSC population (Short *et al.*, 2011; King *et al.*, 2017). The CSC population was also affected by the combination of talazoparib, a PARP1 inhibitor, with IR. The combined treatment induced prolonged G2/M arrest and reduced proliferation rates of GBM CSCs (Lesueur *et al.*, 2018). Altogether, these data suggest that the impairment of repair machinery can sensitize CSCs to IR and could improve therapy efficiency.

Altogether, these observations show the requirement of a more comprehensive analysis of all repair pathways, which can potentially reveal novel opportunities for therapeutic strategies, with either the sensitization of GBM cells to available treatments or the identification of new targets for drug development. It is important to emphasize that resistance is a competence derived from many factors and, besides DNA repair enhancement, drug efflux pumps and alterations in other signaling pathways, as those involved in the control of cell survival, proliferation and apoptosis, surely represent complementary mechanisms of resistance.

## New therapies and future prospective

Researchers have been putting a lot of effort in the development of new therapies and/or approaches that potentially sensitizes GBM to TMZ or IR, as well as in the identification of novel promising drugs. In this ambit, genome-wide and synthetic lethality studies are gaining attention. To enhance efficacy of TMZ, Johannessen and coworkers screened for synthetic lethality using RNAi technology (Johannessen *et al.*, 2019). They used an shRNA library targeting 5,046 human genes to seek for essential genes during TMZ treatment. The knockdown of a 292-gene cluster reduced cell growth of U87 cells when combined with a sublethal dose of TMZ. They also showed that the antipsychotic drug thioridazine mimics the gene cluster silencing, improving TMZ sensitivity *in vitro* and reducing tumor growth *in vivo*. Thioridazine was mechanistically shown to interfere in the autophagy process, impairing the fusion between autophagosomes and lysosomes, and preventing the metabolic adaptive changes related to TMZ resistance in GBM cells (Johannessen *et al.*, 2019).

Other FDA-approved compounds have shown in vitro anti-GBM activity, both isolated and in various combinations. Jiang and coworkers observed that 22 compounds were active against GBM cells. Remarkably, the combination of pitavastatin, used in dyslipidemia control, with low doses of irinotecan, a topoisomerase I inhibitor, showed the greatest potential. Pitavastatin reduced the irinotecan IC50 by 40 to 70-fold (Jiang *et al.*, 2014). Additionally, the repurpose of antihypertensive, beta-blockers (Rundle-Thiele *et al.*, 2016), antidiabetics (Wurth *et al.*, 2013) and anti-alcoholism (Triscott *et al.*, 2012) drugs for GBM adjuvant treatment also demonstrated good potential. As these drugs already have known properties and are approved by drug administration agencies, the clinical application could be facilitated and benefit patients earlier than developing a new drug.

Although studies of new drugs take more time and are more laborious, they are also extremely important and largely conducted worldwide. New drugs for GBM treatment, such as bevazicumab, erlotinib (Raizer *et al.*, 2016), 1,3-bis (2-chloroethyl)-1 nitrosourea (BCNU) (Vinjamuri *et al.*, 2009), and gliadel (Xing *et al.*, 2015), are being massively exploited with good results and some of them have already reached phase II clinical trials. In phase III trials, the implantation of gliadel and BCNU wafers in the after surgery tumor bed was assessed and improved overall survival average in 2 months (Westphal *et al.*, 2003). Sorafenib, a kinase inhibitor that targets multiple RTK receptors, was demonstrated to have *in vitro* and *in vivo* antitumor properties in GBM cell lines (Siegelin *et al.*, 2010). This drug was also able to selectively inhibit GBM CSC proliferation by affecting MAPK and PI3K/Akt pathways (Yang *et al.*, 2010). Sorafenib is under clinical trials to evaluate its safety and efficacy, both as monotherapy and in combination with TMZ, bevazicumab or RT (Wurth *et al.*, 2014).

In addition to the repurpose of FDA-approved drugs and the study of new ones, the development of immunotherapies has also been drawing attention as an important intervention strategy in the last few years. Although pieces of evidence suggest that resistance to radiotherapy can be overcome by immunotherapy in many types of cancer (Gameiro *et al.*, 2014; Maxwell *et al.*, 2017), most clinical trials show no improvement in progression free and/or overall survival in GBM patients (Filley *et al.*, 2017). It is widely known that GBM presents a highly immune-suppressive microenvironment and induces T-cell apoptosis or qualitative defects. Additionally, patients usually present lymphopenia as GBM induces sequestration of T-cells in the bone marrow, reducing their amount at the tumor site and in lymphoid organs; and the blood-brain barrier blocks and/or effluxes antibodies and other large molecules (Sevenich, 2019). Altogether, these features ultimately lead to the poor or no GBM response to immunotherapies observed in clinical trials, e.g., CheckMate 143 (Filley *et al.*, 2017).

However, some strategies have been assessed in order to improve GBM’s response to immunotherapy. For example, Mathios and coworkers showed that local chemotherapy improved the response to anti-programmed cell death protein 1 (anti-PD1) - a checkpoint blockade immunotherapy - in mice by restoring T-cell function and its antitumor activity, while systemic chemotherapy is immunosuppressive (Mathios *et al.*, 2016). Moreover, a recently identified hypermutated GBM subtype, harboring mutations in the exonuclease domain of the polymerase epsilon gene (POLE) and/or biallelic MMR deficiency (bMMRD) (Erson-Omay *et al.*, 2015), respond better to immunotherapies such as immune checkpoint inhibition and neoantigen loads (Bouffet *et al.*, 2016). Hypermutated GBM cells present defective proofreading during DNA replication, which increases mutation rates, stimulating the arising of neoantigens that could activate T-cells and consequently augment the chances of immunotherapy effectiveness (Bouffet *et al.*, 2016). Furthermore, the hypermutated phenotype could also cause the arising of key mutations that provide new tumor competencies, opening the possibility to exploit their synthetic lethality potential and improve treatment response through a combination of radio-chemo-immunotherapy. These evidences highlight the great potential of a combined multi-therapy strategy to overcome GBM’s resistance to the currently available therapies, yet studies in further detail would be necessary to determine the best strategies and protocols.

Moreover, non-classical approaches have been prospected and demonstrated great potential to improve GBM treatment. Tumor treating fields (TTF) is an interesting approach that has been employed in clinics since 2004. TTF modality employs alternating electric fields at an intermediate frequency, from 100 to 300 kHz, to inhibit tumor cell proliferation. It was observed that TTF could suppress invasion and migration of U87 and U373 GBM cell lines, and angiogenesis in endothelial cell lines by downregulation of PI3K/AKT/NF-kB pathway. Impairing of epithelial to mesenchymal transition (EMT) was also observed after TTF, which promoted both increased E-cadherin and diminished vimentin expression (Kim *et al.*, 2016). Additionally, patients treated with TTF presented enhanced overall survival when compared to those who received conventional therapy only (Rulseh *et al.*, 2012).

Another alternative approach of great potential for GBM treatment is the photodynamic therapy (PDT). PDT is based on the administration of a photosensitizing agent (PS), topic or systemically, to the patient with subsequent local exposure to a light source of a specific wavelength, leading to the formation of highly cytotoxic reactive oxygen species. Among its several advantages, worthy of note are the minimum systemic adverse effects due to its double selectivity (de Freitas *et al.*, 2017). In the past decade, the application of PDT to treat GBM has been investigated and proven to be a promising approach, both *in vitro* and *in vivo* (Chakrabarti *et al.*, 2013; Akimoto, 2016). In fact, the Japanese health insurance coverage has introduced, since September 2013, the PDT as a new intraoperative therapy with an indication for malignant brain tumors (Akimoto, 2016).

When applied during surgery, PDT has a double action. Firstly, it helps the surgeon to locate the whole tumor through the fluorescence emission of the photosensitizers (Fluorescence Image Guided Surgical Resection), providing higher rates of complete resection (Eljamel, 2015). Secondly, PDT increases the success of tumor ablation by eliminating its roots while normal brain tissue is spared due to a preferable accumulation of PSs in tumor cells, improving progression-free and overall survival of patients (Akimoto, 2016). However, several variables were found among the studies, including light dose and delivery method, photosensitizer utilized, and sensitivity of different tumor types, evidencing the requirement of standardized clinical trials to effectively evaluate PDT as a treatment option for GBM patients (Quirk *et al.*, 2015). Other modern attempts under study include the use of gamma knife surgery (Skeie *et al.*, 2013), gene therapy (Wilson *et al.*, 2014), modulation of the immune system (Garris and Pittet, 2013), molecular imaging (Jarzabek *et al.*, 2013) and nanoparticles (Tivnan *et al.*, 2017).

## Concluding remarks

Despite all progress made over the last few years concerning both molecular knowledge and novel therapeutic methods, GBM is still poorly responsive to current treatments and extremely lethal. To improve positive impact on patients’ outcome and overall survival, we believe that drug resistance mechanisms and potential novel therapeutic targets should be deeply assessed. Advances in the characterization of CSC are also mandatory for comprehension of resistance and recurrence. To achieve this aim, the understanding of molecular features within the cellular and tissue contexts is imperative. Certainly, each tumor is a unique entity that relies on complex signaling networks and specific competencies working cooperatively to sustain its aggressive phenotype. The research community is only entering a new era of exploring the huge amount of genetic information now available. Hopefully, we expect that in the upcoming years there will be an integrated and meaningful knowledge about the fast progression and high resistance of GBM, allowing the identification of weaknesses of this devastating disease. In this scenario, the appropriate exploration of good molecular targets and adjuvant compounds, allied to refined diagnoses approaches, should permit the development of personalized therapies and establishment of adequate health care for GBM patients.
